# 4349 Survey of Regulatory Reforms to Address Comprehension of Clinical Trial Results

**DOI:** 10.1017/cts.2020.350

**Published:** 2020-07-29

**Authors:** Matthieu Kirkland, Christian Reyes, Nancy Pire-Smerkanich, Eunjoo Pacifici

**Affiliations:** 1 University of Southern California

## Abstract

OBJECTIVES/GOALS: Clinical research is the backbone of the medical community. However, there are few regulations to ensure clinical trial participants can understand their results, leading to volunteers feeling unvalued and unlikely to enroll in trials^1^. This study examines the need of lay summaries METHODS/STUDY POPULATION: To understand the current landscape of clinical trial summaries, literature searches were conducted using the University of Southern California Library database with keywords Title contains “lay language” OR “lay summary” AND any field contains “Trial” OR “clinical”, and Title contains “natural language processing” AND “clinical trial” OR “Summary”. Studies were deemed relevant if they discussed lay language summaries for health care realms or using Natural Language Processing (NLP) to increase comprehension. Papers published by the Center for Information and Study on Clinical Research Participation (CISCRP) were reviewed and their Associate Director was interviewed. RESULTS/ANTICIPATED RESULTS: Of 67 total results, 14 were determined to be relevant. Ten of the relevant results examined lay language summaries and their regulation and 4 were NLP studies. The European Medicines Agency set regulations mandating clinical trial summaries. However, researchers have difficulty validating to an appropriate reading level^2^. Difficulty and potential bias halted a U.S. mandate of lay summaries^3^. The nonprofit CISCRP has partnered with industry to develop unbiased clinical trial summaries resulting in all volunteers feeling appreciated and 91% understanding clinical trial results post summary^1^. Similarly, NLP software for annotating Electronic Health Records increased comprehension for 77% of patients^4^. DISCUSSION/SIGNIFICANCE OF IMPACT: In the U.S., a lack of regulations mandating lay summaries may be related to concerns by regulatory agencies that summaries in plain language may introduce bias^3^. Future looks into integration of NLP systems to clinical trials may create unbiased summaries and allow for FDA regulation.

